# Bevacizumab for paradoxical worsening treatment adjunct in HIV patient with choroidal tuberculoma

**DOI:** 10.1186/s12348-016-0112-1

**Published:** 2016-11-08

**Authors:** Sahil Jain, Priya Bajgai, Basavaraj Tigari, Kusum Sharma, Aman Sharma, Vishali Gupta, Ramandeep Singh

**Affiliations:** Department of Ophthalmology, Advanced Eye Centre, Post Graduate Institute of Medical Education and Research, Sector 12, Chandigarh, India

**Keywords:** HIV-TB co-infection, IRIS, Serous retinal detachment, Bevacizumab, Immune recovery uveitis

## Abstract

The use of anti-tubercular therapy (ATT) along with anti-retroviral therapy (ART) in human immunodeficiency virus-tuberculosis (HIV-TB) co-infected individuals could at times lead to paradoxical worsening due to an increase in the inflammatory activity due to immune reconstitution inflammatory syndrome (IRIS) in the eye. This is characterized by anterior and posterior segment inflammatory reactions which may occur in the form of serous retinal detachment. We describe a case where the use of intravitreal anti-vascular endothelial growth factor (anti-VEGF) agent led to resolution of the serous retinal detachment, which had failed to respond to other common modalities of treatment.

An HIV-TB co-infected 18-year-old, male patient, who was started on ART and ATT developed IRIS in the form of worsening of serous retinal detachment around a pre-existent asymptomatic tuberculoma. The patient was initially treated with oral and topical steroids without a satisfactory response. Intravitreal bevacizumab was then tried for this patient. Serial fundus photos and optical coherence tomography (OCT) taken before and after treatment showed complete resolution of the serous retinal detachment with two doses of intravitreal bevacizumab.

Intravitreal anti-VEGF agents may have a role in the reversal of serous retinal detachment, which occurs as a part of IRIS in HIV-tuberculosis co-infected individuals who have been started on anti-tubercular and anti-retroviral therapies.

## Introduction

TB is one of the most common opportunistic infections in HIV patients; however, ocular TB in HIV patients is relatively rare. It can occur in the form of anterior or posterior uveitis, most commonly in the form of choroidal granulomas [1, 2]. We initiate anti-tubercular therapy (ATT) and anti-retroviral therapy (ART), following which most of the patients improve but some may develop paradoxical reactions to this therapy. Paradoxical reactions are defined as worsening of the disease after starting of the appropriate therapy, which may be seen with the use of either ATT or ART in both HIV positive as well as negative individuals [3–5]. Topical and oral steroids have been often used in the treatment of this condition [6, 7].

We describe a case of serous retinal detachment that developed around an asymptomatic, pre-existent, tuberculoma as a complication of paradoxical worsening of IRIS, who was successfully treated with intravitreal bevacizumab.

## Case report

**Fig. 1:**
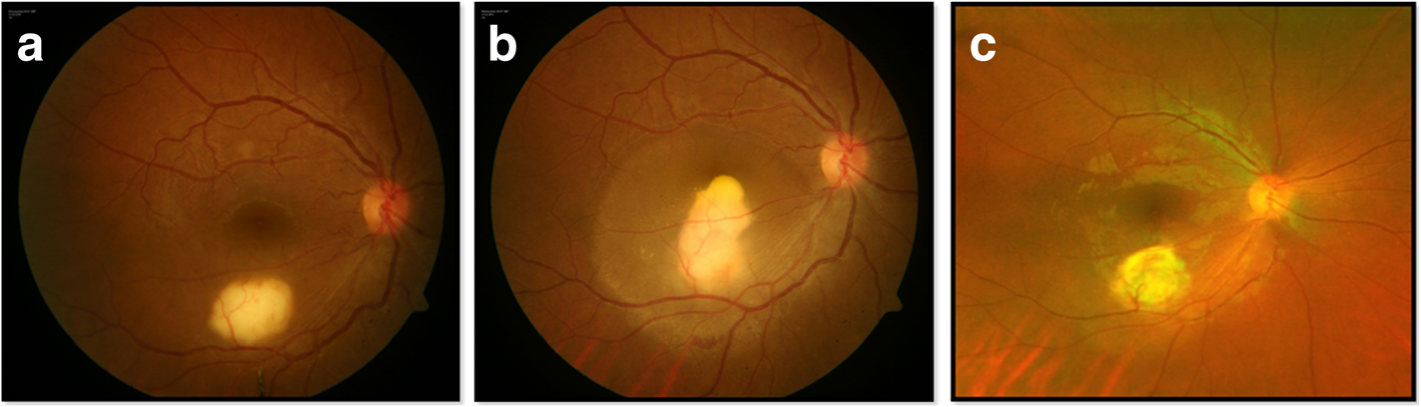
Fundus photograph (**a**) of the right eye at presentation showing a well-defined, choroidal granuloma along the inferior arcade without any fluid around the lesion, (**b**) increase in the size with appearance of surrounding fluid after starting of ATT and ART, (**c**) decrease in the size of lesion as well as of fluid after anti-VEGF therapy

**Fig. 2:**
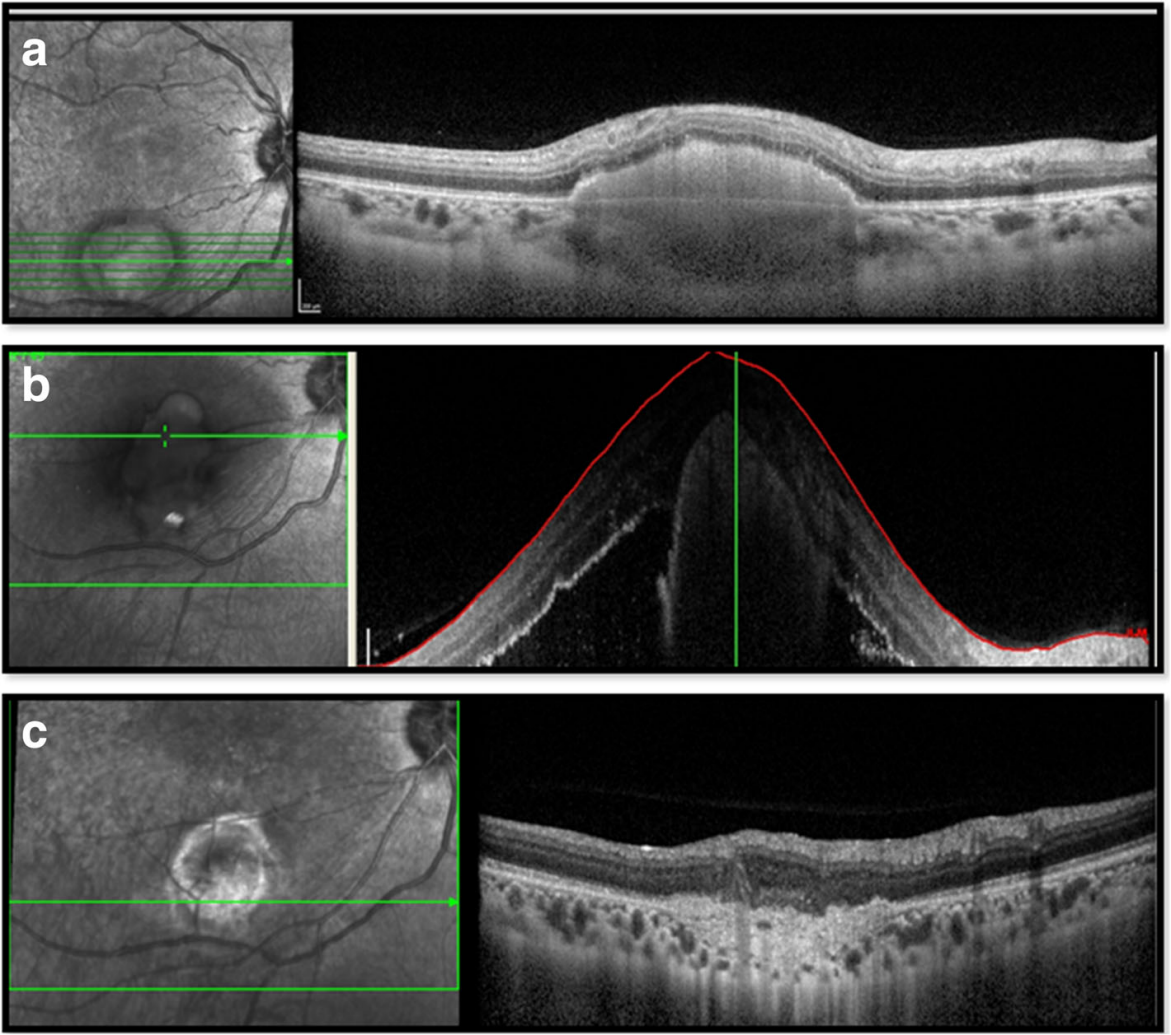
Comparative optical coherence tomography scans passing through the granuloma at presentation (**a**), after starting ATT and ART with massive fluid collection (**b**), and fluid resolution after anti-VEGF therapy (**c**)

An HIV infected 18-year-old, male patient, with newly diagnosed sputum smear negative pulmonary TB and CD4 count of 22 cells/μl presented to us for routine ophthalmic examination, without any ocular complaints. The patient was not on ATT or ART at the time of presentation. On examination, his visual acuity was 20/20 in both eyes, and a quiescent anterior chamber. The fundus examination of the right eye showed a well-defined choroidal tuberculoma of 1.5 disc diameter in size in the posterior pole close to the inferior arcade, not involving the fovea (Fig. 1a) and without vitritis or cystoid macular edema (Fig. 2a). The patient was started on ATT by pulmonologist and 2 weeks later on ART. Five weeks later, he came with complaints of decreased vision in the right eye. His vision was 20/120 in the right eye and 20/20 in the left eye. On examination of the right eye, the patient had mild vitritis along with increase in the size and fluid around the tuberculoma (Fig. 1b). Optical coherence tomography (OCT) showed foveal serous retinal detachment with fluid around the enlarged tuberculoma (Fig. 2b). FFA was done which showed pooling of dye around the lesion in the posterior pole. Internist opinion was sought, and the patient was put on 1 mg/kg oral prednisolone acetate which was given in a tapering dose over a period of 3 months. There was no change in the lesion at 2 weeks. Since there was no improvement, we decided to opt for another mode of treatment in the form of anti-VEGF because of its ability to decrease the vascular permeability. Vitreous tap was taken during injection, which tested positive for TB-PCR.

After two doses of anti-VEGF, the patient had improved visual acuity 20/40. The subretinal fluid had totally disappeared on OCT (Fig. 2c), and there was also a decrease in the granuloma size (Fig. 1c). The patient has been followed up for 12 months following the intravitreal bevacizumab injection, and he has had no fluid or recurrence since then.

## Discussion

Even though HIV infected individuals are at a very great risk for systemic TB, ocular TB is relatively rare. Ocular TB may cause features of anterior or posterior uveitis, amongst which tubercle granuloma is the commonest presentation [1, 2]. ATT is often initiated along with ART to treat the disease, following which most of the patients improve, but some patients may develop paradoxical reactions to therapy [3]. This occurs as the body is able to restore its capacity to mount an inflammatory response against either infectious or non-infectious antigens. The infectious agents implicated are mycobacteria, varicella zoster, herpes viruses, and cytomegalovirus [4].

Ocular paradoxical reactions are associated with decrease in vision, floaters and vitritis, worsening of the original lesion, cystoid macular edema, development of new lesions, and serous retinal detachment as was seen in our patient. CD 4 T cell count has found not to be associated with paradoxical reactions. Corticosteroids have been used to treat the condition [5–7]; thus, we initially tried steroids for him which failed to show any response in our patient. Thus, we sought for an alternative therapy for him. One report [8] has used intravitreal bevacizumab to correct serous retinal detachment, and this also has shown to cause regression of the tubercle granuloma size [9, 10].

Bevacizumab (Avastin; Genentech, Inc, South San Francisco, CA, USA) is a humanized monoclonal antibody against VEGF. We used intravitreal bevacizumab in two doses at 1 month apart for our patient, and there was a marked improvement in both the functional and anatomical outcomes without any deterioration in the next 12 months of follow-up period. In our case, reversal of the vascular permeability by intravitreal bevacizumab played a role in the reversal of the serous retinal detachment. Phase II trials of bevacizumab in HIV patients state that in well-controlled HIV patients, bevacizumab can be given safely, but data on immune-deficient individuals is still awaited and so its use in low CD4 counts remain questionable [11].

Primary treatment for ocular TB remains steroids with ATT in non-HIV patients. But in HIV patients, ATT alone is given without oral steroids and ART is started at 2- to 3-week interval. With the advent of ART, there has been increased incidence of IRIS during the phase of immune recovery. ATT has also solely shown to cause IRIS, even in immune-competent patients. Treatment guidelines for paradoxical IRIS syndrome in HIV are empirical. Our patient developed IRIS in the form of increased serous fluid after being initiated on ATT and ART, and we document its resolution with intravitreal bevacizumab.

## Abbreviations

ATT: Anti-tubercular therapy
HAART: Highly active anti-retroviral therapy
HIV: Human immunodeficiency virus
IRIS: Immune reconstitution inflammatory syndrome
PCR: Polymerase chain reaction
TB: Tuberculosis
